# Impact of post-hatching maturation on the pharmacokinetics of paracetamol in zebrafish larvae

**DOI:** 10.1038/s41598-019-38530-w

**Published:** 2019-02-15

**Authors:** Rob C. van Wijk, Elke H. J. Krekels, Vasudev Kantae, Amy C. Harms, Thomas Hankemeier, Piet H. van der Graaf, Herman P. Spaink

**Affiliations:** 1 Systems Biomedicine and Pharmacology, Leiden Academic Centre for Drug Research (LACDR), Leiden University, Leiden, The Netherlands; 2 Certara QSP, Canterbury Innovation House, Canterbury, UK; 3 Animal Sciences and Health, Institute of Biology Leiden (IBL), Leiden University, Leiden, The Netherlands

## Abstract

Zebrafish larvae are increasingly used in pharmacological and toxicological studies, but it is often overlooked that internal exposure to exogenous compounds, rather than the incubation medium concentration, is driving observed effects. Moreover, as the zebrafish larva is a developing organism, continuous physiological changes impact pharmacokinetic or toxicokinetic processes like the absorption and elimination of exogenous compounds, influencing the interpretation of observations and conclusions drawn from experiments at different larval ages. Here, using paracetamol as paradigm compound, mathematical modelling is used to quantify absorption and elimination rates from internal exposure over time profiles after waterborne treatment, as well as changes in these parameters in post-hatching larvae of 3, 4, and 5 days post fertilisation (dpf). An increase of 106% in absorption rate was observed between 3 and 4 dpf, but no further increase at 5 dpf, and an increase of 17.5% in elimination rate for each dpf. Paracetamol clearance, determined from elimination rate constants and reported total larval volumes of 253, 263, and 300 nL at 3, 4, and 5 dpf respectively, correlates best with higher vertebrates at 5 dpf. This suggests that when studying direct effects of exogenous compounds, experiments with zebrafish larvae are best performed at 5 dpf.

## Introduction

The zebrafish (*Danio rerio*), especially the zebrafish larva, is increasingly used in drug discovery and early drug development, and toxicological screens^1,2^. It is a data and resource efficient vertebrate model organism^3^, that shows 70% genetic homology with humans^4^. Its many advantages include high fecundity and small larval size which is ideal for high-throughput experiments^5^. Additionally, transparency in early life stages enables optical imaging to study *in vivo* effects of exogenous compounds observable by brightfield or fluorescence microscopy. Moreover, it is ethically preferable to perform *in vivo* experiments in the lowest vertebrate, like for example the zebrafish. Additionally, no ethical approval is necessary for studies on larvae before they start independent feeding^6,7^. Experiments in zebrafish larvae bridge the gap between *in vitro* research and *in vivo* preclinical mammal studies as they combine experimental efficiency of cell cultures and organoids with the opportunity to study whole vertebrate organism, including all on- and off-target effects, which will improve extrapolation of observations to higher vertebrates.

In pharmacological and toxicological research with aquatic species, the studied compounds are usually dissolved in the incubation medium (i.e. waterborne treatment). The relationship between the medium concentration of the exogenous compound and its internal exposure is essential for reliable interpretation of the observed results^8–12^, since it is the internal concentration that drives pharmacological and toxicological effects. Because target engagement, which is responsible for the response to exogenous compounds, depends on the pharmacokinetics or toxicokinetics of internal exposure over time, longitudinal data of exposure over time is needed for reliable interpretation of observed effects^13–15^. It is well documented that ignoring this critical issue leads to poor outcomes in drug discovery research^16^.

To derive internal exposure based on the external concentration of the compounds, for example based on physicochemical properties, has been shown to be very challenging^17–19^. Measuring this essential internal exposure is also a challenge due to the small size of zebrafish larvae and the subsequently required very sensitive quantification methods. Recently however, we demonstrated the technical feasibility of measuring pharmacokinetics in zebrafish by developing a profile of internal amount over time for zebrafish of 3 days post fertilisation (dpf), using paracetamol (acetaminophen) as paradigm compound^20^. In this analysis, mathematical modelling was used to describe the pharmacokinetics by quantifying the absorption rate constant, and elimination rate constant which reflects both metabolism and excretion, processes that in addition to the distribution drive the internal exposure.

Although experiments with larva have many advantages, studying an organism during its development will require understanding of the effect of maturation on the studied feature. Zebrafish development is rapid, showing embryogenesis within 3 dpf ^21^, liver budding from 1 dpf and growth from 2 dpf ^22^, development of a functional renal system after 2 dpf ^23^, and presence of a gastro-intestinal (GI) tract from 1–4 dpf ^24^, reaching adulthood in 3 months^25^. These developmental and maturation processes in the first days post fertilisation are expected to have an impact on the absorption and elimination of compounds. As most experiments in the field of pharmacology and toxicology are performed during these first days^1^, it is essential to understand and quantify the impact of development, and to know what difference a single experimental day makes on the internal exposure of exogenous compounds. This is especially the case when studying direct, short-term effects of exogenous compounds.

Using paracetamol as paradigm compound, our aim is therefore to use mathematical modelling to quantify absorption and elimination rate constants from internal exposure over time profiles after waterborne treatment in post-hatching zebrafish larvae of 3 to 5 dpf, and to characterise the impact of development on these parameters using post fertilisation age as marker.

## Methods

### Chemicals

Paracetamol and paracetamol-D4 internal standard were purchased from Sigma (Sigma-Aldrich Chemie B.V., Zwijndrecht, The Netherlands). UPLC/MS grade MeOH was purchased from Biosolve (Biosolve B.V., Valkenswaard, The Netherlands). Purified water (H_2_O) was retrieved from PURELAB (Veolia Water Technologies B.V., Ede, The Netherlands).

### Zebrafish husbandry

All experiments were planned and executed in compliance with European regulation^6^. Handling and maintenance of zebrafish and zebrafish larvae was in accordance with international standard protocols^26^. Adult wild type AB/TL zebrafish were set-up for overnight family cross breeding, separated by sex. Next morning, males and females were combined in breeding tanks with inserts and after 20 minutes eggs were collected. This way, time of fertilisation was controlled. Fertilised eggs were kept at 28 °C in petri dishes in embryo medium (demineralised containing 60 µg/mL Instant Ocean sea salts; Sera, Heinsberg, Germany) which was refreshed daily.

### Experimental design

The experimental design of Kantae *et al*. performed in larvae of 3 dpf ^20^, was repeated with larvae of 4 and 5 dpf in samples of n = 5 zebrafish larvae. In short, two experiments were performed, one in which larvae were continuously treated with 1 mM waterborne paracetamol in embryo medium (treatment medium) for 0–180 minutes and one in which the larvae were treated with treatment medium for 60 minutes and then washed with embryo medium using Netwell inserts filters (Corning Life Sciences B.V., Amsterdam, The Netherlands) and transferred to drug-free medium for a washout period of 60–240 minutes. After the designated constant waterborne treatment or washout period, the larvae were washed with 20/80 MeOH/H_2_O (v/v) using Netwell inserts, transferred to Safe-Lock tubes (Eppendorf Nedeland B.V., Nijmegen, The Netherlands), excess volume was removed and 100 µL 90/10 MeOH/H_2_O with 45 pg/uL paracetamol-D4 internal standard was added. The sample was snap frozen in liquid nitrogen and stored at −80 °C until quantification. Measurements at all time points were performed at least *in triplo*.

Additionally, to ensure paracetamol concentrations in the treatment medium remained constant throughout the duration of the experiment, treatment medium from a set-up with and without 3, 4, and 5 dpf larvae was sampled at 180 minutes and compared to 1 mM paracetamol solution in H_2_O and to fresh treatment medium, all *in triplo*. Samples were frozen at −80 °C until quantification.

### Measurements of internal exposure

The method to quantify internal paracetamol exposure were described earlier by Kantae *et al*.^20^. In short, samples were lysed by iterations of snap freezing the solution with the larvae in liquid nitrogen and submerging the sample in a sonication bath until a homogeneous suspension was obtained. These suspensions were centrifuged at 16,000 g for 10 minutes and 90 µL supernatant was added to 72 µL H_2_O to reach 50/50 MeOH/H_2_O (v/v) to be injected into the ultra-performance liquid chromatography (UPLC) system (Acquity, Waters Chromatography B.V., Etten-Leur, The Netherlands) linked to a quadrupole-ion trap MS/MS (QTRAP, AB Sciex B.V., Nieuwerkerk aan den IJssel, The Netherlands) with an electrospray ionisation source in positive mode. Development criteria were 90–100% accuracy and relative standard deviations less than 10% as measure of precision. Paracetamol concentrations were determined through a calibration curve in matrix ranging from 0.09 to 180 pg/uL, and calculated to total paracetamol amount in pmole per zebrafish larva. Treatment medium samples were diluted with H_2_O to fall within the academic calibration range from 0.05 to 100 pg/uL with a final internal standard concentration of 25 pg/uL paracetamol-D4.

### Pharmacokinetic modelling

To quantify the physiological processes driving the internal exposure of paracetamol, a mathematical model was developed using non-linear mixed effects (NLME) modelling, which combines the quantification of non-random trends in the data called fixed effects as well as random variability known as random effects. NLME modelling was performed using the First Order Conditional Estimation (FOCE) algorithm in NONMEM (v.7.3)^27^, which was operated through the interfaces Pirana (v.2.9.6)^28^ and PsN (v.4.7.0)^29^. Graphical output was generated using R (v.3.4.2)^30^ running through the RStudio (v.1.1.383, RStudio Inc, Boston, Massachusetts, USA) interface.

A one and two compartment model was tested. In case of the two compartment model, the sum of the amounts in both compartments were fitted to the observed total amounts, while elimination was only limited to one compartment. Absorption of paracetamol was estimated as a zero-order process, assuming the paracetamol concentration in the incubation medium to remain constant over time. For the elimination estimation both first-order linear and saturable non-linear Michaelis Menten processes were tested.

Quantification of the residual error was tested as additive, proportional, and a combination of additive and proportional error. As the larvae were lysed to quantify internal exposure, only single observations were obtained from a larval sample. As a result inter-individual variability in internal exposure caused by individual variability in model parameters could not be distinguished from residual variability caused by experimental or analytical error.

Quantification of the correlation between model parameters and larval age, was tested with both continuous (Equations 1 and 2) and discrete (Equation 3) functions:

1 $$P={P}_{base}\cdot (1+slope\cdot (age-ref))$$

2 $$P={P}_{base}\cdot {(1+slope)}^{age-ref}$$

3 $$P=\{\begin{array}{cc}{P}_{1} & age=3\,dpf\\ {P}_{2} & age=4\,dpf\\ {P}_{3} & age=5\,dpf\end{array}$$

where P is the parameter of interest, P_base_ is the base value at the reference age ref, and P_1_, P_2_ and P_3_ are different functions or estimates of the parameter of interest for their respective conditions.

For the continuous relationship, a linear function (Equation 1) or power function (Equation 2) was tested to describe the relationship between age and parameter values. In the discrete function (Equation 3) different parameter values were estimated for larvae older and/or younger than a specified reference age.

For model selection, the likelihood ratio test was used between nested models^31^, assuming a *χ*^2^ distribution and using a significance level of p < 0.01. Additional selection criteria were successful minimisation, estimates of parameter values with 3 or more significant digits and relative standard errors below 50%, and biologically plausibility of the parameter estimates. Graphically, model accuracy was assessed using goodness-of-fit plots, consisting of observed versus predicted plots and conditional weighted residuals (CWRES) versus time or predicted paracetamol amounts, which should show no bias over time or predicted paracetamol amounts^32^. Stability of paracetamol concentrations in treatment medium in the control experiment were normalized to H_2_O control and tested by non-parametric Kruskall-Wallis test, as the data were not normally distributed, with level of significance of 0.05.

### Comparison of paracetamol clearance to higher vertebrates

The degree of correlation between paracetamol clearance in zebrafish larvae with higher vertebrates was assessed by calculating paracetamol clearance values in the larvae by multiplying the obtained elimination rate constants with previously reported total larval volumes at corresponding ages^33^ that are provided in Table 1. This assumes the distribution volume to be equal to the total volume of the larva and a homogenous distribution of the compound throughout the whole larva. The paracetamol clearance values in the larvae at different ages, were graphically compared to reported paracetamol clearance values in higher vertebrates^20^, in a plot of clearance values versus bodyweight of the species. The bodyweight of the larvae was derived from their volume, assuming a density of 0.997 g/mL^20^. A linear least squares regression with 95% confidence interval of the log transformed clearance and log transformed bodyweight was calculated in R, based on clearance values obtained in mature individuals of the different species included in the graph.

**Table 1:** Paracetamol elimination rate constant (k_e_), reported total larval volume^33^, and derived absolute paracetamol clearance (CL) for 3, 4, and 5 dpf larvae.

Age	k_e_ (min^−1^)	Total volume (nL)	CL (nL/h)
3 dpf	0.0193	253	292.3
4 dpf	0.0226	263	356.8
5 dpf	0.0266	300	478.1

## Results

### Measurements of internal exposure

**Figure 1:**
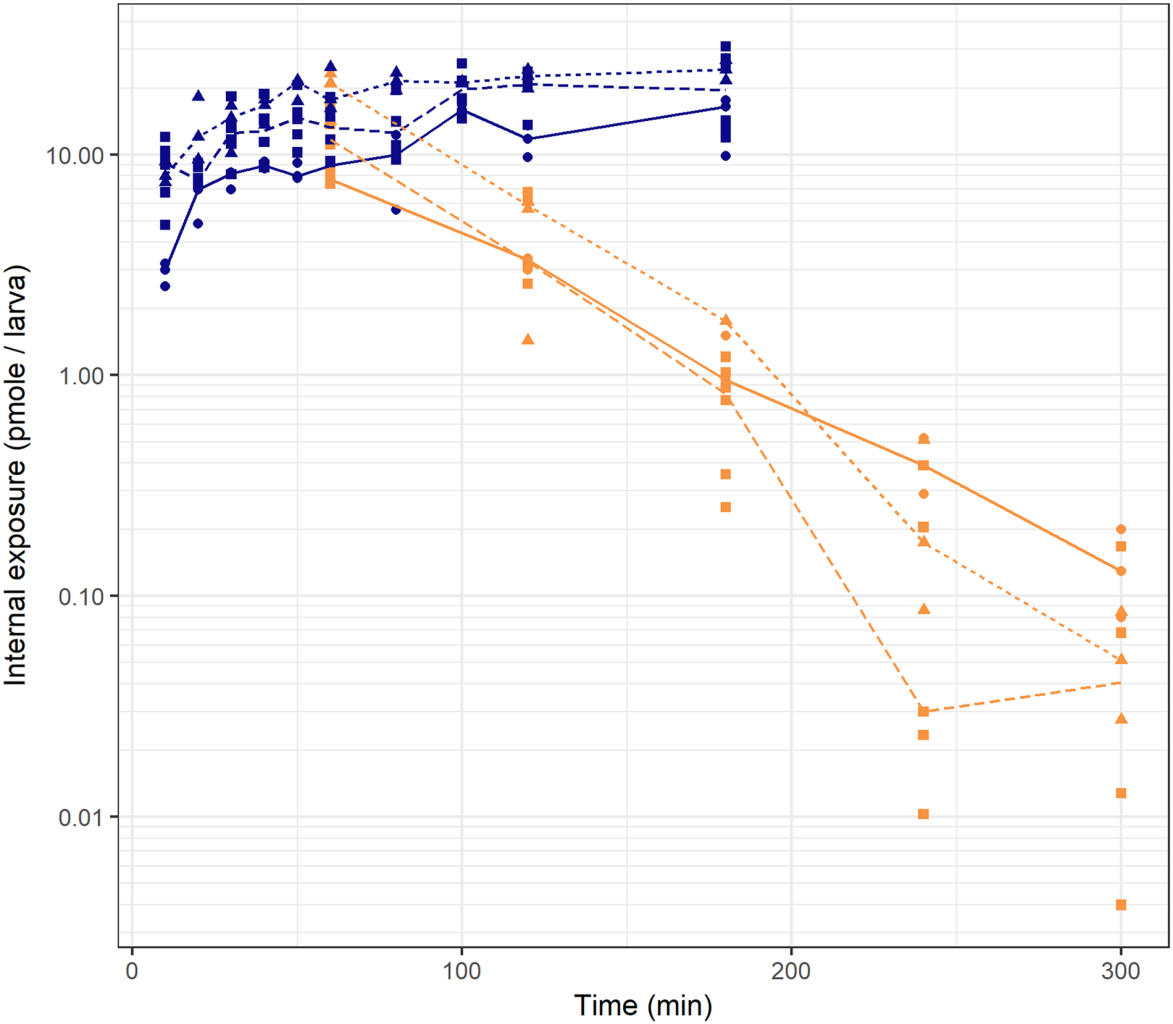
Raw data of internal exposure of paracetamol amounts over time for zebrafish larvae of 3 dpf (solid line, closed circles), 4 dpf (dotted line, closed triangles), and 5 dpf (dashed line, closed squares) for both constant waterborne drug treatment experiment (blue) or washout experiment (orange). Datapoints are total amount per larva from a pooled sample with n = 5. The lines are connecting the median values at each time point.

The observed internal exposure of paracetamol expressed as total amount per larva over time is shown in Fig. 1 for larvae of 3, 4, and 5 dpf for both the constant waterborne treatment and the washout experiment. It can be seen that steady state of internal paracetamol exposure is reached between 100 and 120 minutes of constant waterborne treatment, meaning an equilibrium between paracetamol amounts absorbed and eliminated per time unit has been reached. Steady state exposure in the constant waterborne treatment experiment increased in larvae between 3 and 4 dpf, while it remained relatively constant in larvae between 4 and 5 dpf. The washout experiment showed a mono-exponential decline of the paracetamol amount per larva after the larvae were transferred to paracetamol-free medium. The steepness of this curve, reflecting the elimination rate, increases in larvae with increasing age. The dataset is available through the DDMoRe Repository, Model ID DDMODEL00000294 (http://repository.ddmore.foundation/model/DDMODEL00000294). The stability of the paracetamol concentration in the treatment medium was not impacted by the experimental set-up (Supplementary Fig. 1).

### Pharmacokinetic modelling

**Figure 2:**
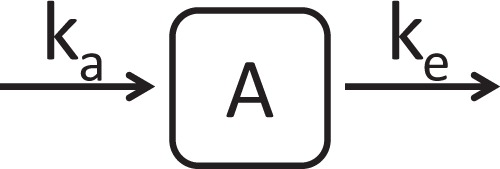
Schematic representation of the final model to describe the total amount of paracetamol in zebrafish larvae over time. ka = zero-order absorption rate constant, A = amount of paracetamol in one larva, ke = first-order elimination rate constant.

Based on the selection criteria, a one-compartment model with zero-order absorption and first-order elimination best fitted the observed profiles of paracetamol amounts in zebrafish larvae over time for both experiments. A combination of additive and proportional error model was found to describe residual variability best, with the variance of the proportional error being 0.109 corresponding to 33% and the variance of the additive residual unexplained error being 0.00844 pmole/larva. A schematic and mathematical representation of this model is provided in Fig. 2 and Equation 4, respectively. The final model included a discrete relationship between age and the absorption rate constant (Equation 5) and a power relationship between age and the elimination rate constant (Equation 6).

4 $$\frac{dA}{dt}={k}_{a}-{k}_{e}\cdot A$$

5 $${k}_{a}=\,\{\begin{array}{ll}{k}_{a,base} & age=3\,dpf\\ {k}_{a,base}\cdot (1+facto{r}_{a}) & 3\,dpf < age\le 5\,dpf\end{array}$$

6 $${k}_{e}={k}_{e,base}\cdot {(1+slop{e}_{e})}^{age-3dpf}$$

where A is the paracetamol amount in a single larva, k_a_ is the zero-order absorption rate constant, k_a, base_ is the base value of the absorption rate constant at the reference age of 3 dpf, and factor_a_ describes the fractional increase in the absorption rate constant in zebrafish larvae that are older than 3 days, k_e_ is the first-order elimination rate constant, k_e, base_ is the base value of the elimination rate constant at the reference age of 3 dpf, and slope_e_ is the estimated slope in the relationships between the elimination rate constant and age.

The obtained parameter values are presented in Table 2 and final model code is available through the DDMoRe Repository, Model ID DDMODEL00000294 (http://repository.ddmore.foundation/model/DDMODEL00000294).

**Table 2:** Obtained model parameter values and their relative standard error.

	Parameter value	Relative standard error (%)
**Structural parameters**		
k_a,base_ (pmole/min)	0.289	4
factor_a_ (−)	1.06	14
k_e,base_ (min^−1^)	0.0193	5
slope_e_ (−)	0.175	18
**Stochastic parameters**		
Variance of proportional residual error (−)	0.109	14
Variance of additive residual error (pmole/larva)	0.00844	48

According to the obtained results, at 3 dpf the value of the zero-order absorption rate constant of paracetamol is 0.289 pmole/min and the first-order elimination rate constant is 0.0193 min^−1^. The absorption rate constant was found to be statistically significantly (p < 1e-10) increased between 3 and 4 dpf by 106% in the final model, but the difference in this parameter between larvae of 4 dpf and 5 dpf was found not to be statistically significant (p = 0.46). The elimination rate constant was found to statistically significantly (p < 1e-6) increase between all three ages. In the final model, the elimination rate constant increased by 17.5% per day, resulting in an elimination rate constant of 0.0226 min^−1^ and 0.0266 min^−1^ for larvae of 4 and 5 dpf respectively.

**Figure 3:**
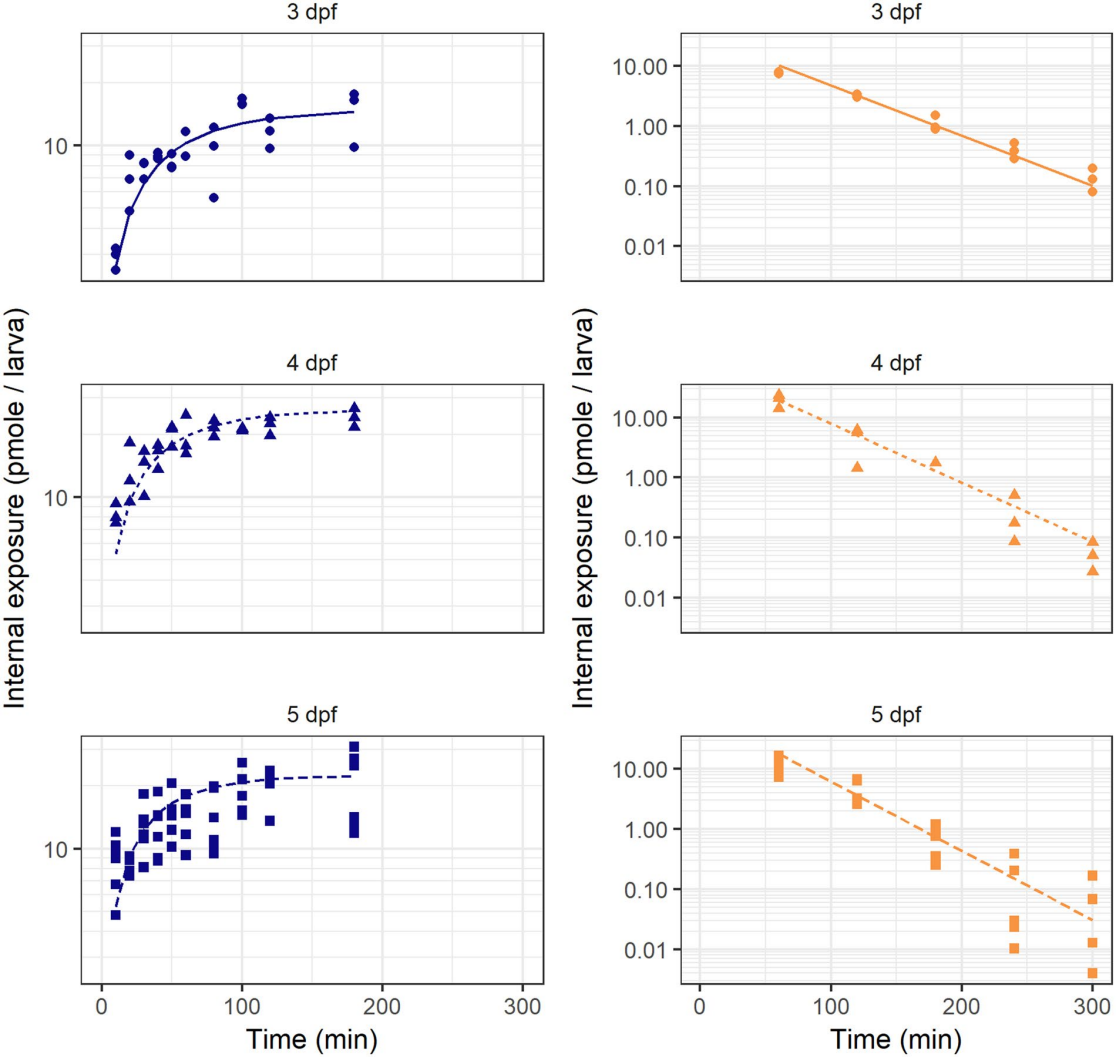
Model fit (lines) through observed paracetamol amounts over time (symbols) in larvae of 3 dpf (solid line, closed circles), 4 dpf (dotted line, closed triangles), and 5 dpf (dashed line, closed squares) for both the constant waterborne drug treatment experiment (blue, left panels) and the washout experiment (orange, right panels).

The model predicted concentration-time profile per age and experiment together with the observed concentrations are shown in Fig. 3, showing good agreement between observed and predicted concentrations. The diagnostic goodness-of-fit plots further confirmed good accuracy of the model predictions (Supplementary Figs 2–4). The relative standard error values of the obtained structural model parameters are well below 20%, indicating good precision of these estimates.

### Comparison of paracetamol clearance to higher vertebrates

**Figure 4:**
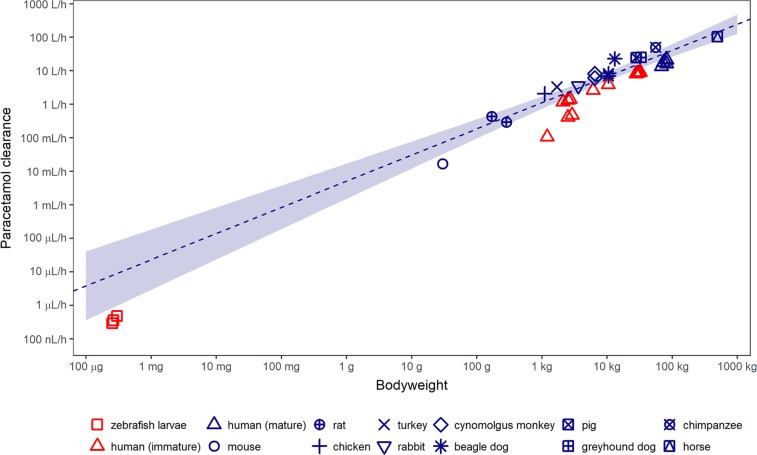
Allometric relationship between paracetamol clearance and bodyweight of 13 vertebrate species including the zebrafish larvae at three different ages. Blue and red symbols show mature or immature individuals of the species, respectively. Allometric relationship (dashed blue line) and 95% confidence interval (shaded area) are determined based on data in mature organisms only. Adapted with permission from Kantae *et al*.^20^.

Paracetamol clearance and previously reported larval volume for 3, 4, and 5 dpf larvae are shown in Table 1. Figure 4 shows the correlation between paracetamol clearance and bodyweight for 13 species including the zebrafish. This plot has previously been reported including the results of zebrafish larvae of 3 dpf only^20^ and now includes also the clearance values for 4 and 5 dpf larvae. It can be seen that the older and heavier larvae show a closer correlation with the higher vertebrates, as they are positioned closer to the 95% confidence interval of the allometric relationship between bodyweight and paracetamol clearance as established based on data from mature individuals only. They do remain below the confidence interval, as do the data points obtained in paediatric human studies (red triangles).

## Discussion

Here the impact of development on the pharmacokinetic or toxicokinetic processes of absorption and elimination through post fertilisation age as marker was quantified by mathematical modelling based on the profiles of internal exposure over time after waterborne treatment in post-hatching zebrafish larvae of 3, 4, and 5 dpf. The absorption of paracetamol was shown to increase 106% between 3 and 4 dpf, but not to significantly further increase at 5 dpf, while paracetamol elimination increased 17.5% per day in this 3 to 5 days post fertilisation period.

Within the mathematical model, the relationships between age and the absorption and elimination rate constants were parameterised with values for larvae at 3 dpf as reference values. These values are comparable to the values reported previously for zebrafish larvae of 3 dpf alone^20^. The doubling of the absorption rate between 3 and 4 dpf, can be explained by the opening of the GI tract, which is a discrete event completing with the opening of the anus at 4 dpf ^7,24^. From that moment, instead of only transdermal or trans-gill absorption, the larvae will also ingest the exogenous compound orally. Recently examined absorption of the antihistamine diphenhydramine between zebrafish embryos and larvae showed an chorion-independent increase in absorption between 2 and 4 dpf and are in concordance with our results here^34^. The absorption rate constant did not increase further between 4 and 5 dpf, suggesting that potential other processes that add to the absorption of paracetamol, do not show maturational changes in the age range studied here.

The 17.5% increase in the elimination rate constant between each of the three post fertilisation days is expected to result from the continuous growth of eliminating organs like the liver and kidneys, as well as continuous maturation of enzymatic processes^22^. Indeed, the clearance values of immature organisms of both the zebrafish and human are lower than expected based on bodyweight alone, but these values do move towards the regression line with increasing age (Fig. 4), with the larval clearance being 35, 41, and 49% of the lower bound of the 95% confidence interval of the extrapolated clearance calculated based on the values of higher vertebrates for larvae of 3, 4, and 5 dpf respectively. It has to be kept in mind that for comparison to higher vertebrates, the absolute clearance in the zebrafish larvae was calculated based on total larval volume, assuming a homogenous distribution over the total body of the larvae, because information on the distribution volume of paracetamol in fish is not available in literature. Given that the distribution volume of paracetamol has been reported to range from 0.8–0.9 L/kg^35,36^ in humans, this assumption seems to be reasonable, although further research into the distribution of this compound in zebrafish larvae is required. If the true distribution volume is larger, the calculated clearance values would also be proportionally larger and fall within the 95% confidence interval, or vice versa. Another factor that may contribute to deviations of the clearance values in zebrafish larvae from the regression line could be the fact that fish are poikilotherms, for which lower metabolic rates have been reported^37^.

From our results it is clear that the age of the larvae during experiments with waterborne treatment influences internal exposure of exogenous compounds, at least for our paradigm compound. Because the internal exposure of the exogenous compound drives its pharmacological or toxicological effect, it can be expected that the age will also impact the observed effects resulting from the treatment. The age of the larvae used to investigate the direct effects of exogenous compounds is therefore an important experimental design consideration. To determine which age to include, three criteria are of importance. Firstly, the internal exposure of the studied compound should be high enough to yield an effect and prevent false negatives. Secondly, the larval metabolic capacity should be large enough to biotransform exogenous compounds to their active metabolites as drug metabolites can also be biologically active. Thirdly, extrapolating observations to higher vertebrates, for instance to improve study design of mammal studies based on translation of clearance, benefits from a direct comparison of the pharmacokinetic processes between species. Based on the results of the paracetamol study presented here, we propose experiments for the testing of direct effects of exogenous compounds to be performed in zebrafish larvae at 5 dpf, because absorption is highest at 4 and 5 dpf, while the metabolic capacity at 5 dpf is largest and clearance at that time resembles clearance of higher vertebrates most within the age range that still falls within the ethical constraints for experiments in zebrafish larvae^6,7^.

## Conclusion

In conclusion, it is of importance to quantify internal exposure over time when testing exogenous compounds by waterborne treatment in zebrafish larvae. The opening of the GI-tract will likely result in increased absorption, which is seen here between 3 and 4 dpf when absorption of paracetamol more than doubles. Continuous growth of eliminating organs as well as maturation of enzymatic processes lead to increased elimination, which is 17.5% daily for paracetamol between 3 and 5 day post fertilisation. To increase internal exposure to parent compounds and metabolites in short term exposure studies, we therefore recommend careful consideration of zebrafish age in experimental design when these pharmacokinetic or toxicokinetic processes are of relevance to the research question. Based on our results with paracetamol, using 5 dpf zebrafish larvae may be preferable for studying direct short-term effects of exogenous compounds.

## Supplementary Information

Supplementary files

Supplementary Dataset 1

## Data Availability

The full dataset and model file are available through the DDMoRe Repository, Model ID DDMODEL00000294 (http://repository.ddmore.foundation/model/DDMODEL00000294).
