# Supplementary material for: Impact of post-hatching maturation on the pharmacokinetics of paracetamol in zebrafish larvae
Source: Sci Rep. 2019 Feb 15;9:2149. doi: 10.1038/s41598-019-38530-w (PMC6377609; doi:10.1038/s41598-019-38530-w)
Supplement: Supplementary file 1 — Supplementary files [file 41598_2019_38530_MOESM1_ESM.pdf]

Impact of post-hatching maturation on the pharmacokinetics of paracetamol in zebrafish larvae

Rob C. van Wijk[1], Elke H.J. Krekels[1], Vasudev Kantae[1], Amy Harms[1], Thomas Hankemeier[1], Piet H. van der Graaf[1,2], and Herman P. Spaink[3*]

[1]Systems Biomedicine and Pharmacology, Leiden Academic Centre for Drug Research (LACDR), Leiden University, The Netherlands; [2]Certara QSP, Canterbury, UK; [3]Animal Sciences and Health, Institute of Biology Leiden (IBL), Leiden University, The Netherlands

[*]Corresponding author: h.p.spaink@biology.leidenuniv.nl

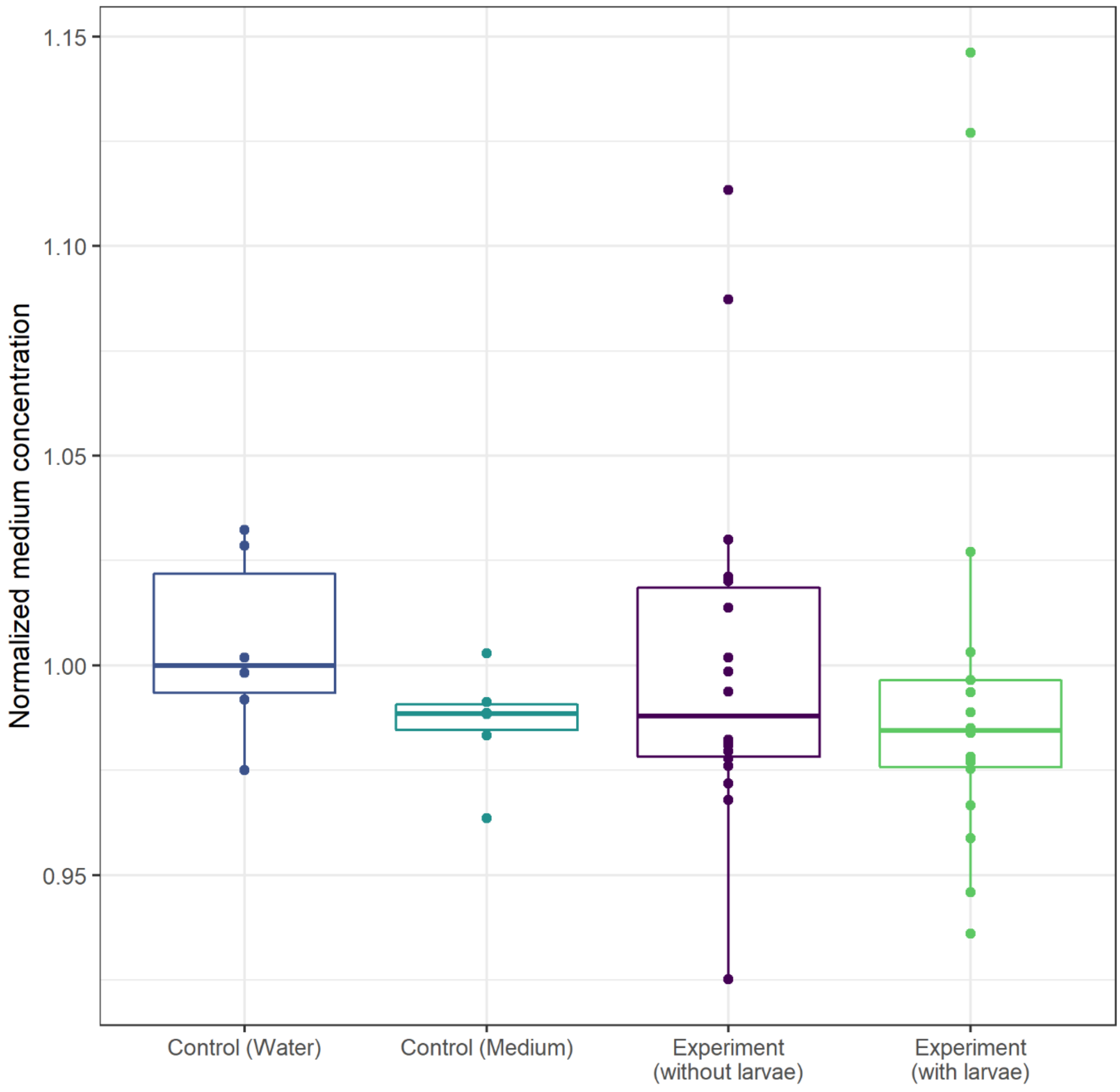

Supplementary figure 1 Control experiment of stability of treatment medium concentration. No statistically significant difference between groups was observed.

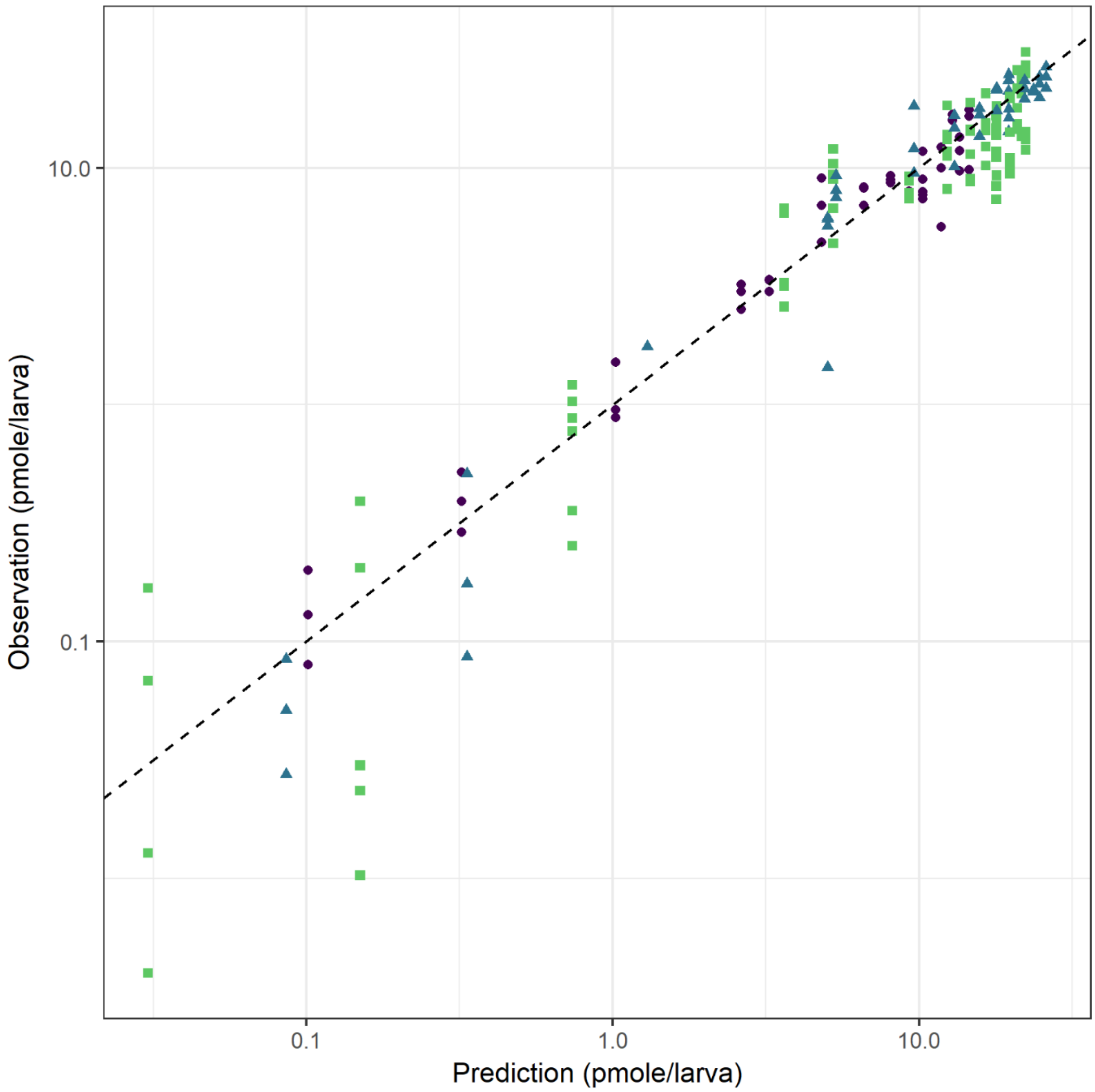

Supplementary figure 2 Goodness of fit plot: Observed vs predicted paracetamol amounts for 3 dpf (●), 4 dpf (▲), and 5 dpf (■) larvae. Black dashed line shows the unity line.

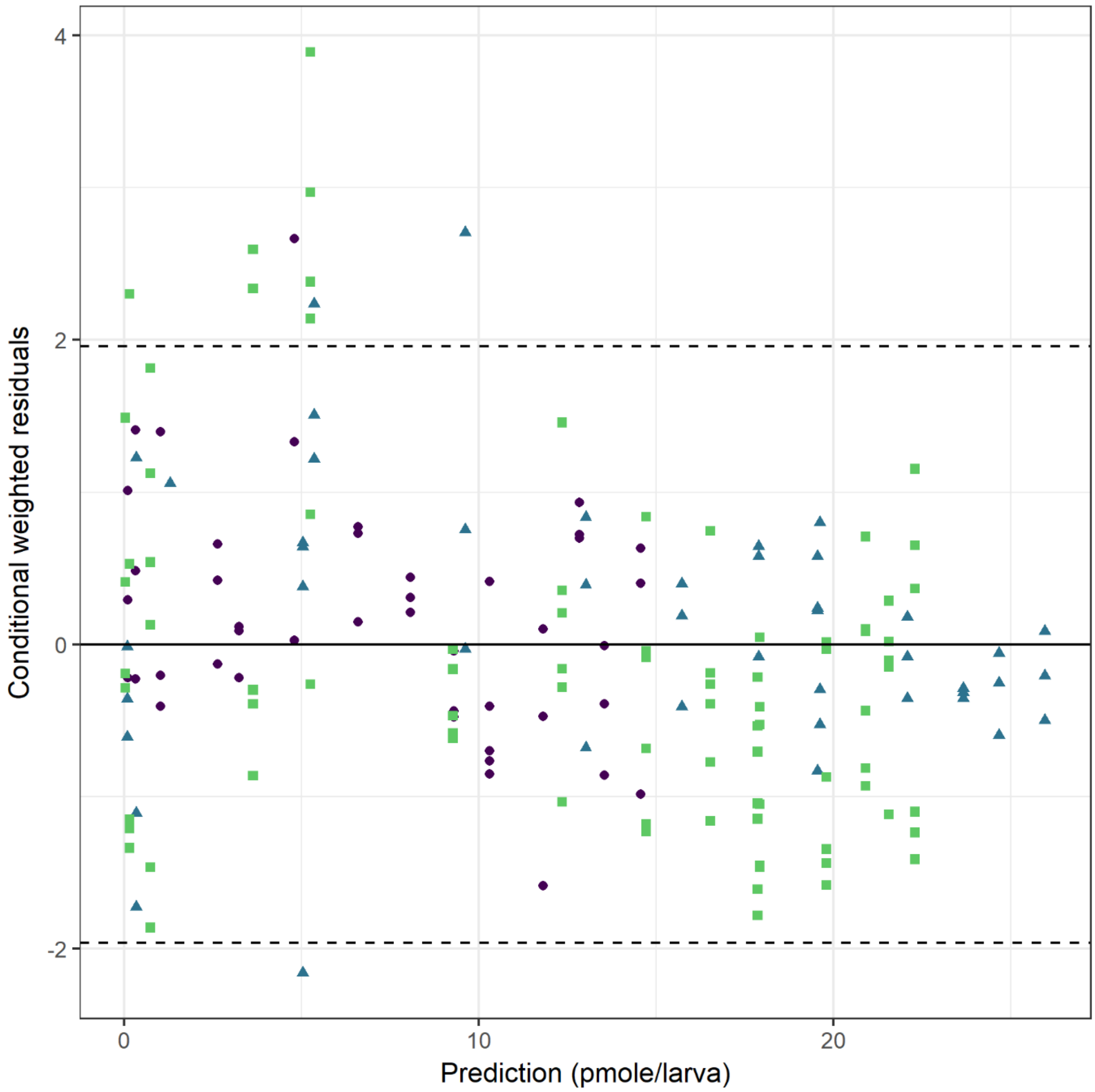

Supplementary figure 3 Goodness of fit plot: Conditional weighted residuals vs predicted paracetamol amounts for 3 dpf (●), 4 dpf (▲), and 5 dpf (■) larvae. Black lines show the line y = 0 (solid) as well as ±1.96 (dashed).

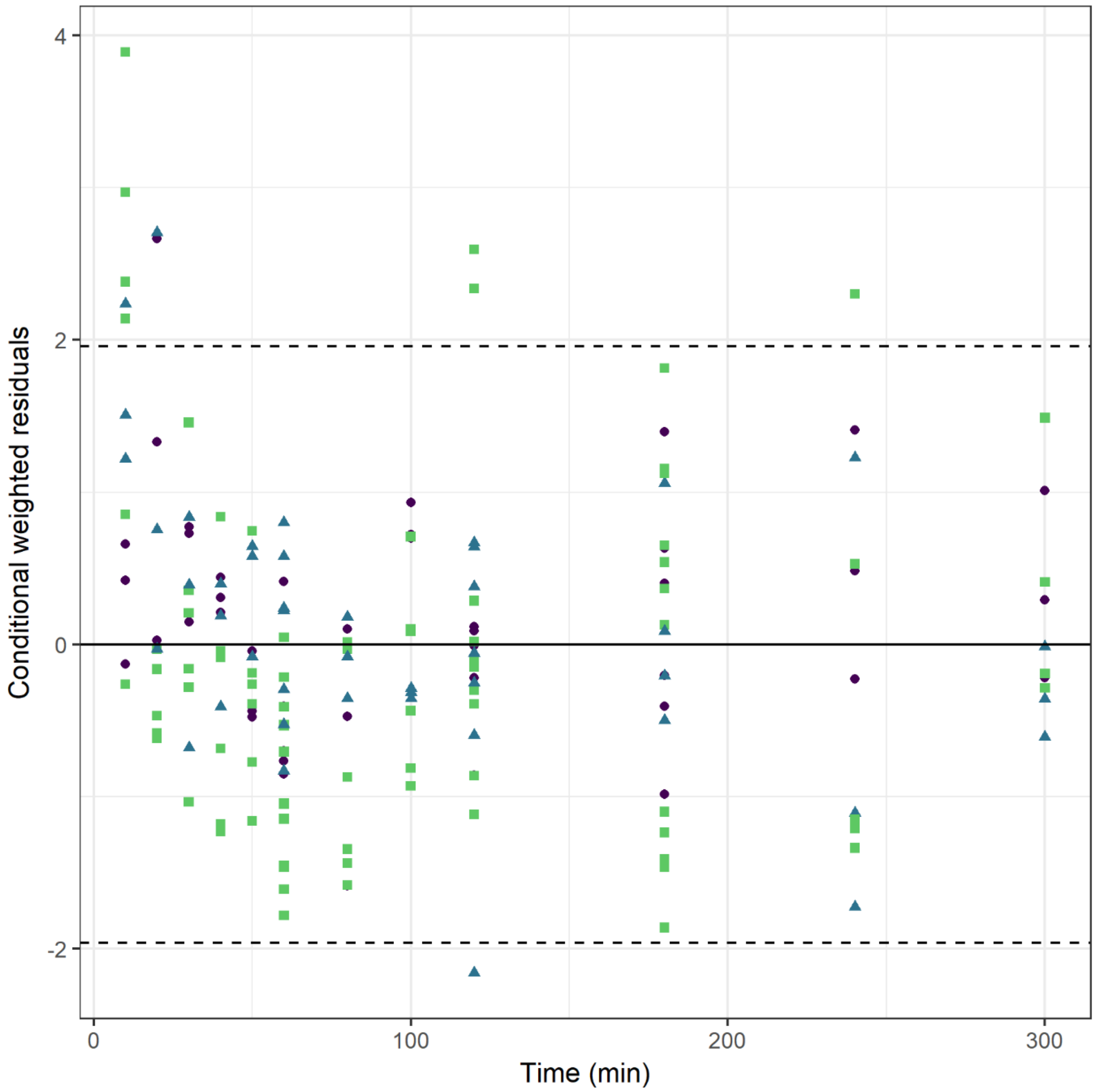

Supplementary figure 4 Goodness of fit plot: Conditional weighted residuals vs time for 3 dpf (●), 4 dpf (▲), and 5 dpf (■) larvae. Black lines show the line y = 0 (solid) as well as ±1.96 (dashed).

The model code (below) and dataset (separate supplementary file) will be online available through the DDMoRe repository, Model ID DDMODEL00000294.

```
;; x1. Author: R.C. van Wijk (r.c.van.wijk@lacdr.leidenuniv.nl)
;; 3. Label: Base and covariate model
;; 4. Dataset: Zebrafish larvae exposed to 1 mM paracetamol at 3, 4, or 5 dpf
;; Description: Paracetamol PK model of 3-4-5 dpf zebrafish larvae, AGE as covariate on KA between 3 and 4dpf, and on K25 for all ages, prop and additive error
$PROBLEM    PK
$INPUT      ID TIME AMT DV EVID MDV CMT BQL AGE
$DATA  Spaink_Impact-of-maturation_dataset_paracetamol_zebrafish_345dpf.csv IGNORE=@ IGNORE=(BQL.EQ.1)

; units
; TIME = min
; DV = pmole / larva
; CL = central volume / min (V = fixed)
; V = total larval volume
; kA = pmole / min

$SUBROUTINE ADVAN13 TOL=9
$MODEL      COMP ; CMT 1 dosing compartment
            COMP ; CMT 2 central paracetamol in larva
$PK
TVK12 = THETA(2)                               ;0-order absorption
IF(AGE.GT.3) TVK12 = THETA(2) * (1 + THETA(3)) ;age-dependent K12 absorption
TVK25 = THETA(1) * EXP(ETA(1))                 ;1-order elimination

K12 = TVK12
K25 = TVK25 * ((1 + THETA(4)) ** (AGE - 3))    ;age-dependent K25 rate of elimination

;base parameters
K25_BASE = THETA(1)
K12_BASE = THETA(2)
;covariate parameters
K12_COVAGE = THETA(3)
K25_COVAGE = THETA(4)
```

```
$DES
DADT(1) = 0 ;constant infusion
DADT(2) = K12 * A(1) - K25 * A(2)

$ERROR
IPRED = F
Y = IPRED * (1 + EPS(1)) + EPS(2) ; prop and add error
IRES = DV - IPRED

$THETA  (0,0.0192529) ; K25
$THETA (0,0.289485) ; K12
$THETA (0,1.06385) ; AGE_K12
$THETA (0,0.174529) ; AGE_K25
$OMEGA  0  FIX  ;    IIV K25, undistinguishable from residual variability
due to destructive sampling
$SIGMA  0.10906  ; prop error
$SIGMA 0.0084383  ;  add error
$ESTIMATION METHOD=1 MAXEVAL=2000 NOABORT PRINT=5 SIG=3 POSTHOC
$COVARIANCE PRINT=E
$TABLE      ID TIME DV IPRED PRED CWRES NOAPPEND NOPRINT ONEHEADER
            FILE=sdtab001
$TABLE      ID K25 K12 K12_COVAGE K25_COVAGE K12_BASE K25_BASE AGE
            NOPRINT NOAPPEND ONEHEADER FILE=patab001
```
